# Pharmacokinetics of Peptide Mediated Delivery of Anticancer Drug Ellipticine

**DOI:** 10.1371/journal.pone.0043684

**Published:** 2012-08-31

**Authors:** Weina Ma, Sheng Lu, Pei Pan, Parisa Sadatmousavi, Yongfang Yuan, P. Chen

**Affiliations:** 1 Department of Pharmacy, No. 3 People's Hospital affiliated to Shanghai Jiao Tong University School of Medicine, Shanghai, People's Republic of China; 2 Department of Chemical Engineering, University of Waterloo, Waterloo, Ontario, Canada; Aristotle University of Thessaloniki, Greece

## Abstract

The amino acid pairing peptide EAK16-II (EAK) has shown the ability to stabilize the hydrophobic anticancer agent ellipticine (EPT) in aqueous solution. In this study, we investigate pharmacokinetics of the formulation of EAK-EPT complexes *in vivo*. The developed formulation can achieve a sufficiently high drug concentration required *in vivo* animal models. The nanostructure and surface properties of EAK-EPT complexes or nanoparticle were characterized by transmission electron microscopy (TEM) and zeta potential measurements, respectively. 12 healthy male SD rats were divided into EPT group and EAK-EPT group randomly. Rats in EPT group were tail intravenously injected with the EPT (20 mg/kg); rats in EAK-EPT group were injected with EAK-EPT complexes (EPT's concentration is 20 mg/kg). EPT was extracted from rat plasma with dexamethasone sodium phosphate as internal standards (IS). The pharmacokinetic parameters were obtained using high pressure liquid chromatography (HPLC). Significant differences in main pharmacokinetic parameters between EPT and EAK-EPT complexes were observed, demonstrating that the complexation with EAK prolongs the residence time of the drug and enlarges the area under the concentration-time curve (AUC). This means that EAK can serve as a suitable carrier to increase the bioavailability of EPT.

## Introduction

**Figure 1:**
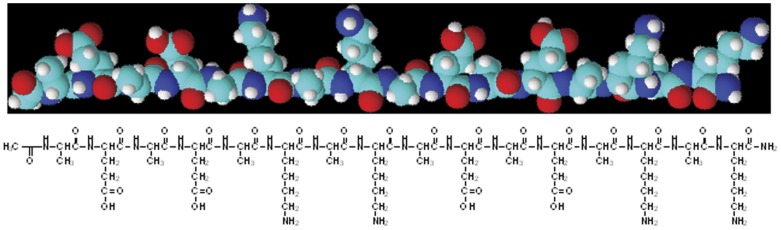
Molecular structures of the self-assembling peptide EAK16-II (EAK).

Self-assembling peptides are emerging as a new type of biomolecules with diverse design potentials and biomedical functions. Among them, a unique class of amino acid pairing, including ionic complementary, peptides have generated much attention owing to their promising performance in drug delivery and tissue scaffolding applications [1]–[2]. Recently, one of the ionic complementary peptides, EAK16-II (EAK), has been found to be able to stabilize the anticancer agent ellipticine (EPT) in aqueous solution [3]–[5]. The peptide is comprised of a 16 amino acid sequence: n-AEAEAKAKAEAEAKAK-c, where A corresponds to alanine, E to glutamic acid, and K to lysine. Acetyl and amino groups protects the N terminus and C terminus of the peptide, respectively. The schematic structure is shown in Figure 1.

**Figure 2:**
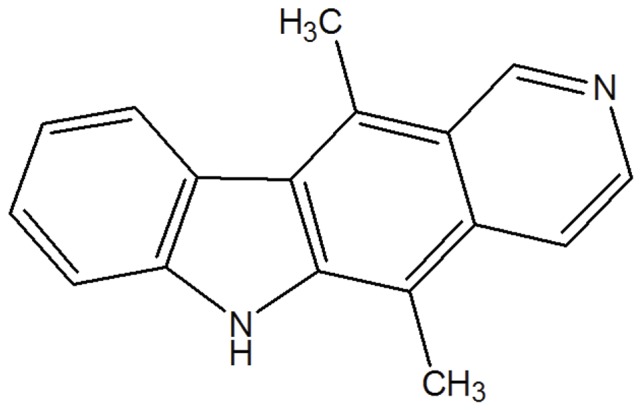
Molecular structure of ellipticine (EPT).

Ellipticine is one of the naturally occurring alkaloids, which is isolated from the leaves of the evergreen tree *Ochrosia elliptica* Labill (Apocynaceae) found in Oceania (Figure 2) [6]. EPT serves as a topoisomerase II inhibitor agent, which can also induce cell apoptosis via p53 and Akt signaling pathways [6]–[8]. It can inhibit osteolytic and breast cancer metastases, kidney cancer, brain tumor and acute myeloblastic leukemia [9]–[10]. In our previous work, the EAK mediated EPT delivery has been extensively studied *in vitro*. As reported, EPT could be stabilized by EAK in aqueous solution with a suitable size distribution in delivery [2]–[3]. The anticancer ability of EAK-EPT complexes/nanoparticles were evaluated *in vitro* on different cell lines [3], [11], with low hemolytic and immune response (unpublished data). In the present work, *in vivo* studies are implemented to evaluate the potential of EAK-EPT systems as an effective therapeutic agent.

This study focuses on investigating the pharmacokinetics of EAK-EPT complexes in plasma following intravenous injection into rats. Healthy male Sprague-Dawley (SD) rats were divided into EPT group and EAK-EPT group randomly. Rats in the two groups were injected with EPT and EAK-EPT complex respectively. Blood samples were collected from orbit. High pressure liquid chromatography (HPLC) was used to detect the concentration of EPT in blood samples. Dexamethasone sodium phosphate was used as internal standards (IS).Effects of self-assembling peptide EAK were investigated on the pharmacokinetics of anticancer drug EPT in rats.

## Results

### Nanostructure of the peptide EAK and EAK-EPT complexes

**Figure 3:**
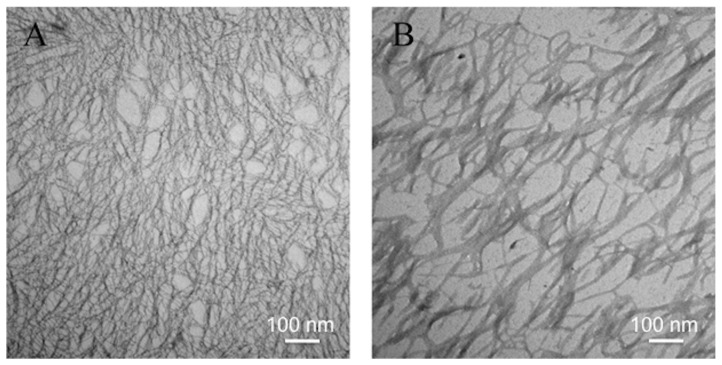
TEM images of (A) EAK16-II, and (B) EAK-EPT. Bar is 100 nm.

EAK16-II has been reported as a self-assembling peptide fabricates fiber nanostructure with a high β-sheet secondary structure. As TEM images (Figure 3A and 3B) showed that EAK, at a really high concentration (3 mg/mL), formed dense fibrous networks; while EAK-EPT showed a less dense network of thicker fibers. The fibrils formed by EAK had the width of around several nm; and the widths of fibers formed by EAK-EPT complexes were estimated to be within the range from ∼20 to 50 nm. The zeta potentials of diluted EAK-EPT complexes samples were measured and listed in Table 1.

**Table 1:** The zeta potentials (mean±S.D., n = 10) of EAK-EPT complexes.

Concentration of EAK in EAK-EPT complexes (mg/mL)*	Zeta potential (mV)
3.0	67.0±5.66
1.5	33.4±3.97
1.0	33.6±3.62
0.5	37.4±3.59
0.3	39.0±4.19
0.1	38.0±4.06

* The mass ratios between EAK and EPT were kept at 2∶1.

### HPLC method validations

#### Selectivity

**Figure 4:**
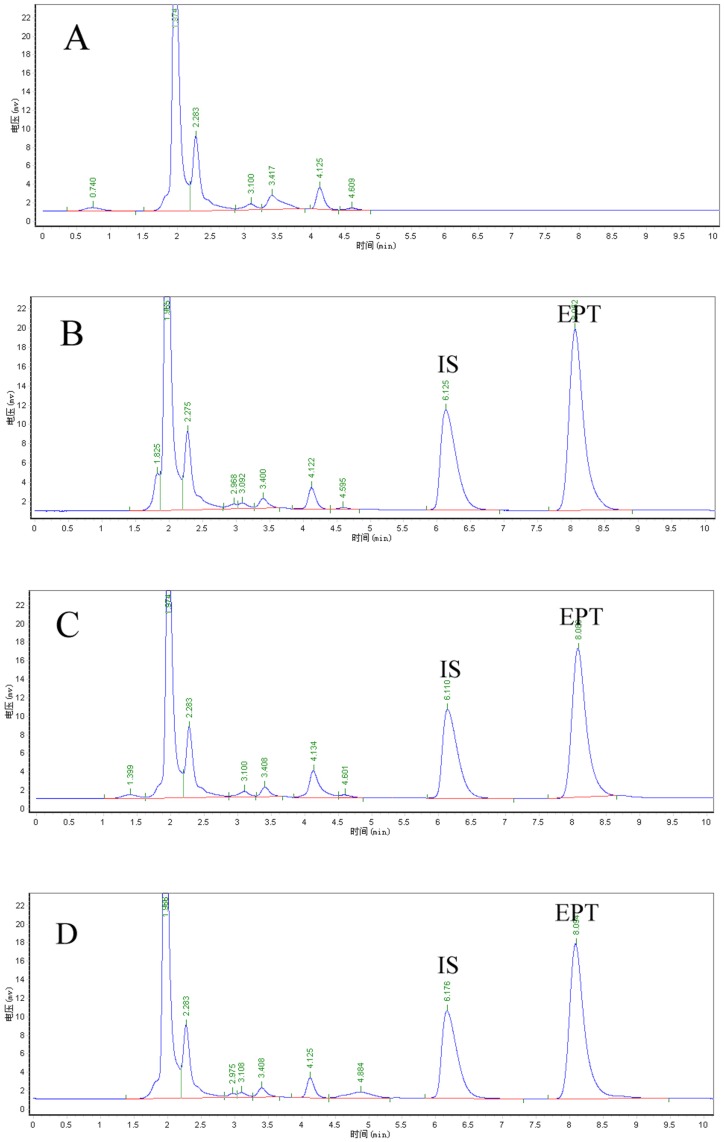
Representative chromatograms of (A) blank plasma; (B) plasma added with EPT (1.56 µg/mL) and IS (dexamethasone sodium phosphate, 2.5 mg/mL); (C) plasma sample after intravenous injection of EPT (3 h); (D) plasma sample after intravenous injection of EAK-EPT (3 h).

EPT peaks were well shaped in both blending EPT plasma samples and endogenous EPT plasma samples. IS (dexamethasone sodium phosphate) peaks were separated from EPT peaks without any interference. The average elution time of EPT and IS was 8.052 min and 6.125 min, respectively (Figure 4). There were no impurity peaks after 5 min in picture A. As showed in picture B, C, and D, the EPT peak and IS peak were separated from the impurity peaks completely.

#### Linearity of calibration curve

The calibration curve was linear over the range of 0.05–6.25 µg/mL. The regression equation of the calibration curve was y = 0.9634x+0.0302, r^2^ = 0.9998 (n = 5). In addition to the plasma concentration of control group at 12 and 24 h, all the data was in the calibration curve range.

#### Precision and accuracy

Table 2 showed the precision and accuracy of EPT QC samples described above. In the measured concentration range, the precision (RSD, %) of intra-day and inter-day ranged from 1.09 to 5.10% and 1.01 to 6.33%, respectively; while the accuracy (%) of intra-day and inter-day ranged from 99.17 to 106.23% and 98.94 to 109.14%.

**Table 2:** The validation of intra- and inter-day precision and accuracy with EPT QC samples (n = 5).

Concentration (µg/mL)	Precision (RSD %)		Accuracy (%)	
	Intra-day	Inter-day	Intra-day	Inter-day
0.05	5.10	6.33	106.23	109.14
0.39	2.32	2.07	102.71	104.26
3.13	1.09	1.01	99.17	98.94

#### Recovery

The mean recoveries of 5 repeated EPT QC samples for each of the three concentrations, 0.05, 0.39 and 3.13 µg/mL were 91.49±5.08, 96.60±3.20, 102.41±2.34%, respectively.

### Pharmacokinetic studies of EPT and EAK-EPT

**Figure 5:**
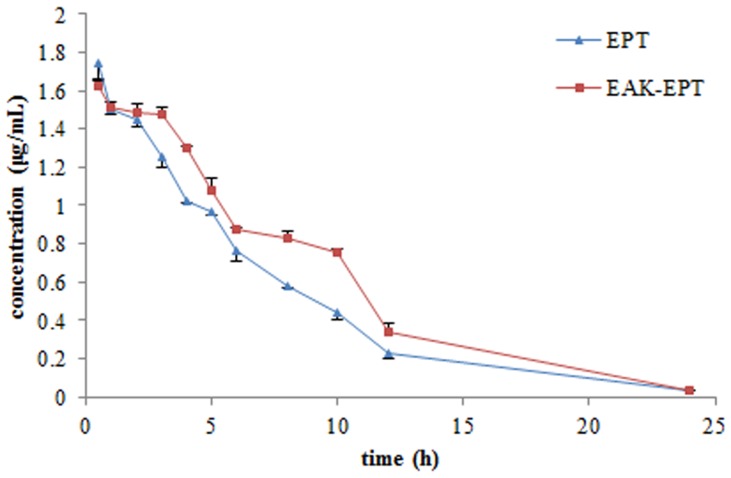
Mean plasma concentration-time profiles of EPT in SD rats (n = 6) after single intravenous injection of EPT and EAK-EPT at a dose of 20 mg of EPT per kg of body weight.

Plasma concentration-time curves of EPT were shown in Figure 5. The relevant pharmacokinetic parameters, including t_max_, C_max_, T_1/2α_, T_1/2β_, CL, MRT and AUC, were listed in Table 3. Compared to the EPT group, EAK-EPT group has higher concentration of EPT in plasma from 0.5–24 h, and the AUC was significantly increased (p<0.01). The T_1/2α_, T_1/2β_ and MRT of EAK-EPT also increased significantly (p<0.01); inversely, the C_max_ and CL of EAK-EPT were both lower than the EPT group (p<0.05 and p<0.01). T_max_ of EPT group and EAK-EPT group were both 0.5 h.

**Table 3:** Pharmacokinetic parameters (mean±S.D., n = 6) of EPT after single intravenous injection of EPT and EAK-EPT at a dose of 20 mg of EPT per kg of body weight.

Parameters	EPT	EAK-EPT
C_max_ (µg/mL)	1.75±0.10	1.63±0.04*
t_max_ (h)	0.50±0.00	0.50±0.00
T_1/2α_ (h)	4.50±0.71	6.69±0.27**
T_1/2β_ (h)	4.75±0.18	6.71±0.26**
CL (mL/h)	1.38±0.03	1.09±0.02**
MRT (h)	5.78±0.10	6.24±0.13**
AUC (h⋅µg/mL)	12.29±0.27	14.89±0.36**

* p<0.05 compared with EPT group;

** p<0.01 compared with EPT group.

## Discussion

According to the TEM images, the nanofibers of EAK-EPT complexes, after formulating with EPT, were significantly thicker compared with EAK self-assemblies. This increase in the widths of EAK-EPT nanoparticles compared to that of EAK self-assemblies was probably due to the entrapment of protonated EPT (Fluorescence results indicated that EPT was in protonated state. Data was not shown) between peptide nanofibers/molecules. The size of a drug delivery system is always an important criterion to evaluate the potential as a therapeutic agent. The nanostructure of EAK-EPT co-assemblies, as shown in Figure 3B, was dendritic: the spread narrow fibers interflowed at one end to form a “trunk”. Most of the “trunks” had the width of ∼50 nm and the length from 100 nm to 200 nm, which is a suitable size for therapeutic nanoparticles.

Zeta potential is also an important element to judge the stability of nanoparticles. As shown in Table 1, the zeta potential of EAK-EPT nanoparticles, after 30 times dilution, was still above 30 mV, indicating that the structure of nanoparticles were very stable. This favorable stability would enforce the absorption of active forms and prolong the residence time of the nanoparticles even after long time circulation in blood.

Through the process of HPLC detection, drug extraction from plasma samples was the most time-consuming and tricky step [12]–[14]. Conventional extraction agents included ethyl acetate, methanol and mehyl tert-butyl ether (MTBE), which were tested in preliminary experiments. MTBE was found to induce high noise, while low recoveries were found when ethyl acetate and methanol were used to precipitate the plasma protein. Compared with the conventional extraction agents, an extraction agent composed of 50 µL 1 M Na_2_HPO_4_ and 800 µL acetonitrile showed better capabilities towards the precipitation of plasma proteins, as well as on extracting EPT from plasma samples. This is probably due to the fact that Na_2_HPO_4_ could increase the pH of plasma, resulting in deprotonation of protonated EPT in plasma samples. The generated neutral form of EPT is hydrophobic, which facilitated the extraction of EPT from the plasma to the acetonitrile phase. The HPLC assay was then performed by using a modified mobile phase containing 1% KH_2_PO_4_ aqueous solution and Acetonitrile (70∶30, v/v), in pH of 5.0, at the flow rate of 1.0 mL/min. All the elution was finished within 10 min, and EPT peak was separated from the IS peak and impurity peaks completely within the time period.

According to the results of the HPLC method validation and material selection, it suggested our method could provide quick quantified analysis with high precision. Moreover, the high method recovery rate indicated that the method for plasma sample pretreatment was effective and accurate. The method of sample preparation can extract EPT from plasma, as well as separate EPT from plasma proteins and other impurities successfully.

The results of pharmacokinetic study showed that the nanoparticle EAK-EPT and the prototype drugs EPT were significantly different in bioavailability. As shown in Table 3, the AUC of EAK-EPT group, comparing with that of EPT group, increased more significantly, indicating enhanced bioavailability of EPT after formulating with EAK. The changes of ETP concentrations in plasma exhibited different behavior in EPT groups and in EAK-EPT groups. Specifically, the decrease of EPT concentrations, in EAK-EPT group, in plasma over time was slower than that in EPT control group. To our knowledge, prototype EPT has a strong metabolism in the intestines and liver, which can be quickly eliminated from blood [15]. After formulating with EAK, the EPT was protected by peptide molecules with suitable nanoparticles sizes, as well as good stability. These favorable properties most likely facilitated the transportation of EPT through the blood vessels and slowed down the liver metabolic rate on EAK-EPT nanoparticles. All the results presented show that the amino acid pairing peptide, EAK-16 II, could enhance the bioavailability of prototype drug EPT and prolong the residence time of EPT *in vivo*.

## Conclusions

In this study, an effective high pressure liquid chromatography method was developed for the determination of the anticancer drug ellipticine (EPT) carried by an amino acid pairing peptide, EAK, in an animal model. This method was successfully applied to the pharmacokinetic analysis of EAK-EPT complex/nanoparticle formulation in rats. The EAK-EPT complexes, prepared with a 2∶1 mass ratio, had a favorable size distribution for therapeutic nanoparticles; the pharmacokinetic results showed that the EAK-EPT formulation could prolong the residence time of EPT and enlarge the area under concentration time curve (AUC). Compared with the EPT control, EAK-EPT nanoparticles had a significantly higher bioavailability. These results indicated that the peptide EAK had the potential to deliver and enhance therapeutic efficacy of EPT in the future clinical application.

## Materials and Methods

### Reagents and chemicals

EAK16-II (1657.66 g/mol, >95% pure by HPLC) was purchased from CanPeptide Inc. (Pointe-Claire, Quebec, Canada). The anticancer drug ellipticine (98% purity) was purchased from EMD Biosciences (Canada). Dexamethasone sodium phosphate was purchased from The National Institute for the Control of Pharmaceutical and Biological Products (Shanghai, China). Acetonitrile, phosphoric acid and Dimethyl sulfoxide (DMSO) were purchased from J&K Chemical Ltd. (Shanghai, China).

### Formulation of EAK-EPT complexes

The complexes were made of EPT (1.5 mg/mL) with freshly prepared EAK solution at concentration of 3 mg/mL to obtain a 2∶1 mass ratio of EAK to EPT. A certain amount of EPT powder was first weighted and added into freshly prepared EAK solution (the pure water was filtered through 0.2 µm filter); then the suspension was mixed vigorously by vortexing for 24 hours, the resulting solution is cloudy; afterwards, 1 M HCl was added into the solution to adjust the pH until the solution got clear (to make all the EPT in protonated form); and then, 1 M NaOH was added into the solution to adjust the pH back to 5.6–5.8. The samples for zeta potential measurements were prepared by diluting the complexes (3 mg/mL EAK with 1.5 mg/mL EPT) in pure water with pH adjustment to ∼5.6. A control sample was also prepared by dissolving EPT powder in 5% ethanol aqueous solution. HCl and NaOH were added to adjust the pH to be consistent with complex sample.

### Transmission electron microscopy (TEM)

Images were obtained using a Philips CM10 TEM. A volume of 10 µL of peptide or peptide-drug complexes was incubated on the grid (Canemco & Marivac,Canada) for 10 seconds, and stained with uranyl acetate/lead citrate (Electron Microscopy Sciences).

### Zeta potential

The zeta potentials of peptide-drug complexes were obtained on a Zetasizer Nano ZS (Malvern Instruments, Worcestershire, U.K.). The appropriate settings, viscosity, refractive index, and dispersant solvent, were set for each measurement at 25°C. A volume of 700 µL of the sample was transferred from the vial to a clear disposable zeta cell (Malvern Instruments, Worcestershire, U.K.). The data was obtained and analyzed by software package Dispersion Technology Software 5.1 (Malvern Instruments, Worcestershire, U.K.).

### Ethics Statement

All the procedures and care administered to the animals have been approved by the institutional ethic committee, under a permit of animal use (Approval ID:2011-024) in the No. 3 People's Hospital affiliated to Shanghai Jiao Tong University School of Medicine, compliance with the Experimental Animal Regulation by the national Science and Technology Commission, China.

### Animal model

Healthy male Sprague-Dawley (SD) rats, 6 week old and 180–220 g, were obtained from the B&K Universal Group Limited (Shanghai, China). The rats were maintained under a 12 h light/dark cycle at 25°C and a humidity of 60±10%. The animals were allowed to acclimatize for 2 weeks after arrival. The total 12 rats were divided into two groups randomly: EPT group and EAK-EPT group. Each animal fasted overnight with free access to water before drug administration. Rats in EPT group were tail intravenously injected with the EPT (20 mg/kg); the rats in EAK-EPT group were injected with EAK-EPT complexes (EPT's concentration is 20 mg/kg). After administration, 300 µL blood samples were collected from orbit at the time points of 0.5, 1, 2, 3, 4, 5, 6, 8, 10, 12, 24 hours, respectively. The blood samples were centrifuged at 5000 rpm for 10 min to obtain plasma samples. All the plasma samples were stored at −80°C before HPLC analysis.

### HPLC assay

#### Chromatography system

The chromatography system used was composed of a Shimadzu LC-10AT vp series chromatographic system (Shimadzu, Kyoto, Japan) with SPD-10AT vp UV detector. Data processing was performed with LC Solution software. Analysis was carried out by using Dikma Dimosil C-18 column (150 mm×4.6 mm i.d., from Dikma Technologies, China). The mobile phase was consisted of a mixture of 1% KH_2_PO_4_-Acetonitrile (70∶30, v/v) and regulated pH to 5.0 by phosphoric acid. After degassing by ultrasonication the mobile phase was delivered at a flow rate of 1 mL/min. The column effluent was monitored by UV detection at 300 nm.

#### HPLC sample preparation

Stock solution (1 mg/mL) of EPT in DMSO: Acetonitrile (1∶9, v/v) were stored at −20°C. The working solutions of EPT at concentration of 31.25, 15.63, 7.81, 3.91, 1.95, 0.98, 0.49, 0.24 µg/mL were prepared by serial dilutions of EPT stock solution with the same solvent. 100 µL blank plasma was obtained by centrifugation of orbital blood; then 20 µL EPT working solutions and 50 µL IS (dexamethasone sodium phosphate, 2.5 mg/mL) were respectively spiked into the blank plasma; 50 µL 1 M Na_2_HPO_4_ and 800 µL acetonitrile were added to remove the plasma protein. The blood samples (100 µL) were also mixed with 50 µL IS solutions, 50 µL Na_2_HPO_4_ and 800 µL acetonitrile to remove the plasma protein. After centrifuging at 5000 rpm for 10 min, the supernatant acetonitrile was collected and dried by nitrogen gas. The dried samples were re-dissolved in 100 µL mobile phase and vortexed. Afterwards, the mixtures were centrifuged at 10,000 rpm for 10 min; 20 µL of the supernatants were injected into the HPLC for detection.

#### Calibration curve

Eight EPT working solutions with different concentrations were prepared as described in section HPLC sample preparation. The peak areas of dexamethasone sodium phosphate (A_D_) and EPT (A_E_) were recorded, and the values of A_D_/A_E_ and the concentrations of EPT were used to plot the calibration curve (y = ax+b).

#### Accuracy and precision

EPT quality control (QC) was performed by using EPT working solutions with the concentrations of 0.05, 0.39 and 3.13 µg/mL, respectively. The preparation of blank plasma followed the steps in section HPLC sample preparation. The intra-day accuracy was determined by assaying five replicates of each QC sample at 0, 2, 4, 6, 8, 10 h in one day; inter-day assay accuracy and precision were determined by assaying five replicates of each QC sample on day 1, 2, 3, 4, 5.

#### Methods recovery

EPT and IS working solutions were added into the blank plasma at QC levels, and then the QC samples were prepared by using the method described in section HPLC sample preparation. The peak area ratio (A_D_/A_E_) was compared to that for EPT and IS in DMSO –acetonitrile solution at the same concentration.

#### Pharmacokinetic study of EPT and EAK-EPT

Standard methods were used to calculate the following pharmacokinetic parameters using non-compartment analysis (WinNonlin software version 4.1; Pharsight Corporation, Mountain View, CA, USA): total area under the concentration time curve (AUC), time-averaged total body clearance (CL), mean residence time (MRT), the distribution half-time (T_1/2α_), and the elimination half-time (T_1/2β_). The peak concentration (C_max_) and the time to reach peak concentration (t_max_) of EPT were determined directly from the experimental data.

#### Statistical analysis

The results were expressed as mean values ± SD. C_max_ and t_max_ were tested by non-parametric tests (Wilcoxon signed rank test). The other data were analyzed by t-test or t'-test. P values<0.05 were considered to be statistically significant.
